# Artificial Womb Technology and the Choice to Gestate *Ex Utero*: Is Partial Ectogenesis the Business of the Criminal Law?

**DOI:** 10.1093/medlaw/fwz037

**Published:** 2019-12-18

**Authors:** Elizabeth Chloe Romanis

**Affiliations:** Department of Law, Centre for Social Ethics and Policy, School of Social Sciences, University of Manchester, Manchester, UK

**Keywords:** Abortion, Artificial wombs, Ectogenesis, Miscarriage, Pregnancy, Termination of pregnancy

## Abstract

It is frequently claimed that artificial wombs (AWs) could alleviate the burdens placed exclusively on women in reproduction. In this article, I demonstrate how AWs used for the partial gestation of preterm neonates could introduce new choices for women by changing perceptions of tolerable risks in gestation. In light of advancing medical technology, it is necessary to consider whether the current legal framework in England and Wales would support increased choice for women about alternative forms of gestation. I examine the ill-defined offence of ‘unlawfully procuring miscarriage’ in the Offences Against the Person Act 1861 and demonstrate that different conclusions about the legal significance of ending a pregnancy are evident, depending on the analytical lens adopted in interpreting ambiguities. Furthermore, I demonstrate that the defences available to pregnancy termination under the Abortion Act 1967 are too narrow to support choices about alternative forms of gestation, even if they become physically and medically possible. Therefore, we should decriminalise termination of pregnancy, or, if it is assumed that gestation is the business of the criminal law, specific reforms to the legal framework are necessary. The offence of unlawfully procuring miscarriage is too uncertain and broad, and the defences available are too restrictive.

## I. INTRODUCTION

In 2017 and 2019, two teams of researchers claimed that their prototype artificial womb (AW) devices, successfully tested on animals, might soon be ready for use on humans.^1^ It is thought that AWs can better guarantee that preterm delivery does not *necessarily* result in death/severe complications. Moreover, because the technology is designed to continue the process of gestation *ex utero*^2^ (this process is known as partial ectogenesis),^3^ AWs could become a reliable alternative to later term pregnancy. There is general consensus that AWs should be welcomed both to aid preterms^4^ and to alleviate women^5^ of the burdens placed solely on them in reproduction.^6^ It has been posited that AWs could reduce the burdens placed exclusively on women in reproduction, because the technology might reduce the need for pregnancy.^7^ In this article, I demonstrate how artificial womb technology (AWT) will change how risk is perceived and assessed during pregnancy. AWs could present an empowering choice for some women: enabling them to choose an alternative to pregnancy without risking the loss of their future child. This choice could be sought by women for a variety of reasons, but would be particularly empowering for women experiencing dangerous yet wanted pregnancies. The availability of AWs in place of neonatal intensive care (NIC) would shift perceptions of risk in pregnancy, potentially resulting in an increase in terminations of pregnancy that are not intended to result in foetal death. There is, therefore, a need to consider the legalities of opting for *ex utero* gestation.

The clinical translation of prototype AW devices into treatment for human neonates is fraught with ethico-legal issues.^8^ In this article, I do not deal with these questions but rather focus on technical legal questions that AWs will bring. It should be noted, however, that pregnant women will necessarily shoulder any risks associated with AW development. Thus, that women may not necessarily be as emancipated by AWT as has been suggested,^9^ because of potential legal restrictions, is all the more concerning. Examining the extent to which the current legal framework is compatible with future reproductive technologies is essential, because AWs bring new challenges concerning their accessibility and application.^10^ While aspects of my analysis are unavoidably speculative, it is important to raise these questions before the first human use of AWs.^11^ Moreover, this investigation allows for a meaningful critique of current provisions. The Abortion Act 1967 (AA 1967) has frequently been criticised for affording doctors, rather than women, the power to make choices about women’s bodies.^12^ In light of emerging technologies, it remains important to continue the debate about whether the medical monopoly on termination decisions is appropriate. Such analysis can meaningfully add to the contemporary debate about decriminalisation.

Scholarship exploring the ethico-legal implications of AWs has been primarily directed towards the potential movement of the viability threshold, and thus whether there will be,^13^ or should be^14^ more limitations on access to conventional abortion (a termination of pregnancy resulting in foetal death).^15^ In this article, I do not raise such questions because AWs do not present an alternative to conventional abortion. First, ‘foetal extraction’ for gestation *ex utero* would be a much more invasive procedure than a conventional abortion^16^ (drug-induced or vacuum/surgical). Secondly, AWT will not be able to sustain embryonic products of conception in the foreseeable future, and most women seeking conventional abortion opt for abortion before 13 weeks.^17^ Finally, the claim that AWT is an ‘alternative’ to abortion fundamentally misunderstands why abortion is protected in liberal societies,^18^ including women’s experiences and motivations in seeking abortion.^19^ For these reasons, it is important that we preserve liberal access to conventional abortion.

In this article, I argue that partial ectogenesis to allow a woman who might otherwise have to continue a pregnancy experiencing some burden (or have an unwanted conventional abortion) should be lawful. I argue that pregnant women, rather than medical professionals, should be empowered to determine what threshold of risk in their pregnancy justifies foetal transfer to an AW. I demonstrate in this article, however, that present law does not accommodate this possibility. Thus, we should either decriminalise terminations of pregnancy in England and Wales, or reform current legal provisions on the grounds that they are too broad, and the circumstances in which terminations are lawful are too restrictive. It is clear that the criminal law, in particular offences so old and out-dated, should not be the basis of medical regulation in this matter. However, since pregnancy termination remains a criminal offence, I offer an account of the problems the current legal framework creates. I limit my discussion to partial ectogenesis^20^ and to situations in which women^21^ want to end their pregnancies without corresponding foetal death.

First, I outline the prospects for, and potential uses for, AWs in place of neonatal intensive care. Secondly, I outline the potential impact of the technology on perceptions of risk in pregnancy and demonstrate how AWs have particular utility in minimising the burdens women have to experience in pregnancy, especially in those instances in which pregnancy is, or becomes, dangerous. Thirdly, I outline the criminal law surrounding termination of pregnancy and some of the inadequacies in the regulation, particularly with regard to the application of the legal framework to emerging biomedical technologies. It may be surprising to many that in 2019 the termination of pregnancy remains a criminal offence in England and Wales.^22^ The Offences Against the Persons Act 1861 (OAPA 1861)^23^ established the offence of unlawfully procuring miscarriage that remains the basis of the law today, 158 years later. The offence is committed when a pregnant woman, or any other person, takes steps to unlawfully procure miscarriage with the intention of procuring miscarriage.^24^ A pregnant woman is only guilty if she is actually pregnant at the time of the attempt,^25^ whereas other persons can be found guilty of unlawfully procuring miscarriage irrespective of whether the woman is pregnant.^26^ Medical professionals are afforded a defence in England and Wales under particular circumstances prescribed in the AA 1967. Despite the offences contained in the OAPA 1861 forming the foundations of the law, I demonstrate that the terminology used to describe the offence is vague. There is no definition of ‘miscarriage’ in any criminal statute. Miscarriage was interpreted to mean ‘the termination of an established pregnancy’ in a high court challenge concerning the legality of contraceptives.^27^ This is so broad, however, that it becomes confusing when applied to decisions made later in pregnancy. The definition does not illuminate whether ‘miscarriage’ encompasses any deliberate ending of a pregnancy, or only those resulting/intending to result in foetal death. I demonstrate that there are plausible different interpretations that might be afforded to ‘unlawful miscarriage’, such that there are different legal consequences to opting for *ex utero* gestation depending on the analytical lens that is adopted in interpreting the OAPA 1861. Moreover, the use of the phrase ‘unlawfully’ in the OAPA 1861 itself means that the parameters of the offence are ill-defined. If a doctor terminates a pregnancy outside of the specific defences granted in the AA 1967, but with the intention of ‘treating’ the delivered foetus in an AW, have they committed the offence of *unlawfully* procuring a miscarriage?^28^ Answering this question is crucial because it establishes whether all medical interventions to end pregnancy are in need of legal justification, or only a subset of terminations with particular consequences. Finally, I consider the defences available in the AA 1967 for doctors providing terminations of pregnancy with the intention of continuing gestation *ex utero*. I argue that the AA 1967 does not account for shifting perceptions of risk that AWs could bring because it does not provide permission to end a pregnancy for less severe risks or non-medical reasons, *even if* the foetus survives the termination. This analysis exposes how restrictive the AA 1967 is.

## II. THE PROSPECTS FOR AWT

In 2017, a US research team announced the development of the ‘biobag’.^29^ This prototype AW had, in early trials, successfully supported lamb foetuses on the viability threshold for 4 weeks.^30^ In 2019, a second research team in Australia published the results of an alternative design named the ‘EVE platform’ with some comparable success.^31^ These devices are designed to replicate the conditions and function of the human uterus so that the developing human entity subject to the device (the gestateling)^32^ is able to continue to gestate. The gestateling is submerged in artificial amniotic fluid in a sealed plastic bag. Circulation is maintained by the gestateling’s own heartbeat (assisted by an oxygenator) and catheters imitating umbilical cord access. These studies signal a change in approach: from assisting a preterm with the necessary bodily functions for survival in the external environment that they are attempting, but struggling, to perform themselves, to continuing the process of gestation *ex utero*.^33^

Prematurity remains the leading cause of death for infants born at or before 24 weeks,^34^ because the infant is usually too functionally immature^35^ to survive, even with assistance. In addition to the innate risks of mortality and morbidity in prematurity, the use of conventional NIC technologies also harbours significant risk of serious injury or death due to infection,^36^ irreversible lung damage,^37^ and heart failure.^38^ The biobag demonstrates the opportunity to deal with both of these problems. The lamb subjects in the trial all exhibited organ maturation and growth.^39^ Moreover, there were no incidences of infection or heart failure during the experiment and, after the experiment, all subjects were delivered from the AW ‘healthy’ with normal heart and lung function.^40^ Similar results^41^ were reported with the EVE platform, leading the research team to conclude that their findings demonstrate ‘the potential clinical utility of a further refined EVE therapy system to improve outcomes for extremely preterm infants’.^42^ The biobag team suggests that, following further scientific and safety validation,^43^ they will soon start preparing for testing on humans.^44^

It is initially anticipated that these AW devices could aid gestatelings removed from the uterus at, or just below, the borderline of viability (23–24 weeks).^45^ Current methods of NIC have succeeded in pushing back our understanding of viability,^46^ but this appears to have reached a natural limit at 22 weeks.^47^ Resuscitation, and/or subsequent support provision, is usually not given to preterms delivered before 22 weeks,^48^ because these preterms are thought to be too developmentally immature to benefit.^49^ In theory, AWs would not be subject to the constraints of gestational maturity.^50^ Therefore, if AWT is used to sustain ‘just-viable’ preterms successfully by imitating gestation, there could eventually be instances where the technology is used to sustain younger preterms. Doctors will have clinical incentives to attempt to use the technology at the request of women^51^ to support preterms delivered before the current threshold.^52^ Over time, it is plausible that the technology could increasingly be used to sustain less functionally mature developing human entities, for example, gestatelings removed from the uterus as young as 18 weeks.^53^

In the animal studies discussed, the subjects were transferred into the AW devices after caesarean delivery. As a result, attempts were made to control the stress experienced by the subject in transfer. For example, a drug was administered to the subject to ensure that fluid did not drain from the lungs.^54^ In addition, the subjects did not experience any of the potential pressures or trauma in a natural birth. For this reason, I envisage these AW devices being most effective in situations where delivery can be managed by caesarean, rather than following spontaneous preterm deliveries. This is not to say that attempts would not be made to utilise AWs as treatment in those cases, only that there is cause for speculation that outcomes might not be as promising. For the purposes of this article, those instances in which preterm delivery is anticipated and is actively undertaken are of more interest.

## III. CHANGING CHOICES IN OBSTETRICS

AWs could empower women who struggle to make decisions about pregnancies that have become, could become, or are perceived to be, dangerous. Moreover, AWs could provide women with an increased ability to decide what risks they are willing to assume during gestation. In this section, I demonstrate a potential shift in risk perception that occurs with the availability of AWs, and how this might lead to a change in demand (both in nature and frequency) for endings to pregnancy. The choice to end a pregnancy and deliver a foetus preterm (before 37 weeks)^55^ is a difficult, but not uncommon, choice that women and obstetricians must navigate when pregnancies go wrong. This choice (to induce a premature end to the pregnancy) is a response to complications that threaten the life or health of the pregnant woman or her foetus. Examples of cases in which premature ending of pregnancy is considered necessary to manage risks in a pregnancy include: placental abruption,^56^ severe traumatic injury,^57^ preeclampsia,^58^ chronic hypertension,^59^ diabetes,^60^ unmanaged uterine infection,^61^ significant foetal compromise,^62^ maternal cancer, and foetal growth restriction.^63^ Combined, these complications in pregnancy are not uncommon, and therefore the induced ending of a pregnancy to manage them is also not uncommon.

The decision about whether a pregnancy should be continued, or ended either by a termination likely to result in foetal death, or by ‘premature delivery’ with the intention that the delivered preterm will be sustained with NIC, depends on a number of factors that have to be balanced. The crucial relevant factors include: the gestational age and maturity of the foetus, the severity (and nature) of the complication, additional risks increasing with the duration of pregnancy, the risk for the foetus *in utero*, and the risk to the foetus of being born premature.^64^ The removal of the foetus before complete gestation is risky because the foetus is underdeveloped^65^ and usually cannot survive without support. NIC can improve the chances of survival, but there remains a high risk of complications^66^ causing disability or death.^67^ As gestation advances, the threshold of risk of early delivery falls,^68^ because the likelihood of complications resulting from premature birth decreases with gestational age^69^ and maturity.^70^ Where the pregnant woman is stable enough and anxious to save her foetus, termination is undertaken as late as possible. Ending the pregnancy is seen as a last resort, because NIC is no guarantee that a delivered preterm can survive.

Once a decision is made to end a pregnancy, and the timing decided, the mode of ending the pregnancy must be determined. The mode of termination will determine the outcome. In what are thought of as ‘attempted premature deliveries’, the foetus is extracted from the uterus by caesarean (the surgical opening of the abdomen and womb)^71^ or drug-induced vaginal delivery.^72^ These methods of ending pregnancy carry their own risks.^73^ Deciding if, when, and how to end a pregnancy is a case of balancing the risks affecting the pregnant woman and the foetus in continuing the pregnancy, versus ending it. In some cases, when continuing the pregnancy endangers both the pregnant woman and the foetus, opting to end the pregnancy (and provide NIC post-birth) can be a clear decision to make in order to attempt to save both. This decision can be difficult in cases where continuing with the pregnancy is dangerous for the pregnant woman, but would be beneficial for the foetus, and termination carries greater risks for it. In deciding whether to continue or end the pregnancy, such risks can be hard to weigh both for doctors exercising clinical judgement, and for pregnant women anxious for their own health and well-being, and for their foetuses. This decision-making calculus, however, could become less challenging with AWs.

Used as an alternative to conventional methods of NIC, AWs have the potential to produce better and more consistent outcomes for developing human entities removed from the uterus before 37 weeks. Current NIC is seen as an unreliable/dangerous alternative to remaining pregnant (because of the inherent risks and limitations) for pregnant women who are concerned with not losing their foetuses. Most pregnant women who seek to end their wanted pregnancies before full term do so *only* because their pregnancies are dangerous, and they seek to remain pregnant for as long as possible because of their concerns about their foetuses and prematurity/conventional NIC. AWs, however, could eliminate much of the concern over the risks of prematurity, and the limited success of NIC. This could enable a new kind of decision-making in obstetric practice, shifting the focus to the woman’s experience of pregnancy, rather than the ‘foetus’s interests’ in not being removed from the uterus prematurely. AWs thus have the potential to enhance women’s autonomy by lessening the pressure incumbent on them to act in self-sacrificial ways during a wanted pregnancy. This change in focus could result in more ‘endings of pregnancy’ being considered clinically appropriate, and/or endings of pregnancy on medical grounds being advised earlier in gestation. This could then reduce the pressure on women in terms of forcing them to make difficult decisions about termination themselves.^74^ AWs have the potential to become not just about rescuing developing human entities from the effects of prematurity, but also about rescuing pregnant women from aspects of pregnancy without requiring women to sacrifice or risk their foetuses. There are four ways in which AWs will reframe perceptions of risk in pregnancy.

First, taking the case of dangerous pregnancies, it is possible that AWs will shift perceptions of what level of risk would be sufficient to justify an intervention to end a pregnancy on the grounds of a woman’s health. At present, the concerns about prematurity mean that only the most serious risks to the pregnant woman’s health and life are considered medically sufficient grounds to end pregnancies prematurely. This often results in women making the choice to sacrifice their own health.^75^ If there were a more reliable alternative to natural gestation than conventional NIC, there is every possibility that the threshold of sufficient risk to justify terminations will be lowered. This might be lowered to include less severe complications (such as the earlier stages of preeclampsia or less serious traumas) that are not thought sufficiently grave to justify risking a woman’s wanted foetus being born premature, but might be thought sufficient to justify ending a pregnancy in favour of artificially continued gestation. Pregnant women concerned for their foetuses would consequently no longer have to be advised on the basis of needing to tolerate the same level or degree of risk. Moreover, lesser dangers to their health and well-being will be considered more serious.

Secondly, AWs could impact on perceptions of viability and could, therefore, remove the importance placed on gestational maturity in obstetric decision-making. If foetuses are considered ‘viable by virtue of technology’^76^ earlier in a pregnancy, this could diminish the emphasis placed on the timing of delivery in the decision-making process that obstetricians evoke when considering bringing a high-risk pregnancy to an end. With the concern about foetal viability increasingly removed from the equation, and because lower levels of risk that signal the need for intervention are likely to occur earlier in pregnancy, there could not only be an increase in premature endings to pregnancy, but these terminations could be more ‘premature’. There might be attempts to transfer foetuses to AWs very early in gestation (18 weeks), but also more routine attempts to end pregnancies closer to the threshold of viability (22–24 weeks) as opposed to continuing and monitoring the pregnancy to ensure delivery occurs as far along as possible.

Thirdly, if AWs become reliable, obstetricians would not have to frame their advice to pregnant women as a ‘balancing act’ in terms of weighing up the risk to their foetuses against the risks to their health in remaining pregnant. Presently, even when a woman’s health is at risk due to her pregnancy, she is often advised (by choice) about the severity of the risk to her, *in comparison to* the severity of the risk to her wanted foetus of terminating early. Often women are advised to end pregnancy only when the threat to their health is serious, because only this is thought to *outweigh* the risk to the foetus of prematurity/NIC. If AWs were available, and could mitigate the impact of prematurity, less emphasis would be placed on a comparative account of risk. Whilst it could never be guaranteed that there is no risk to the foetus in removal for gestation *ex utero*, there would not be the *same* impetus to evaluate risks to the foetus. If the technology were considered reliable, any risks to the foetus would result only from the process of transfer, as opposed to the risks of preterm delivery coupled with the inherent risks of being born premature. With less concern regarding the impact of prematurity on the part of both pregnant women and their obstetricians, it becomes possible for the nature of the discussions regarding management of dangerous pregnancies to shift. Instead of a comparative account of risk to the pregnant woman and to her foetus, conversations could be directed more towards the pregnant woman’s condition in itself, and the associated risks for her in continuing the pregnancy. The pregnant woman would be free to make decisions predominantly on the basis of her own health with the knowledge (if her concern) that prioritising her health will not always be in direct conflict with the foetus's welfare. The technology will have the potential to change the language used in these circumstances of the ‘maternal-foetal conflict’,^77^ a common conceptualisation that is the result of the pregnant woman and foetus’s physical interests being understood as in competition with each other.

The decision about whether to end a pregnancy *for medical reasons* could be reduced to a case of considering the impact of pregnancy on a woman’s health, and weighing up the risk to her health of termination against remaining pregnant and delivering at the end of 37 weeks. In most cases, especially when a woman’s life or health is at risk, these risks are likely easily resolved because the mode of delivery would be the same whether the pregnancy is delivered preterm, or at term (by caesarean). Arguably, a managed elective caesarean is likely to be less risky for women in such cases than an emergency caesarean.^78^ Even if this were not the case, such a balancing exercise would more often be resolved in favour of termination when there is any risk in remaining pregnant to a woman’s life or health. I will return to the issue of balancing the risk of termination versus the risk of remaining pregnant in legal terms later in this article.

Finally, if AWs were a reliable alternative to pregnancy a demand might emerge for endings to pregnancy (in favour of *ex utero* gestation) in less urgent or in non-medical circumstances. Pregnant women whose pregnancies pose a lesser risk to health may request to opt for an alternative to their gestation. Unpleasant or uncomfortable, but not actively dangerous, experiences during pregnancy might encourage women to seek termination in favour of AWs. Unrelenting morning sickness, mobility issues and swollen limbs, migraines, insomnia, anxiety, fear of developing post-partum depression, and plenty of other side effects can be difficult to endure for some women. Kendal suggests that there could also be women who seek termination in favour of an AW to evade social stigmas associated with pregnancy.^79^ For example, women concerned about the impact on their work and potential discrimination or women struggling with addiction.^80^ There could, in these cases, be innate risks apparent in the preterm extraction of a foetus for *ex utero* gestation that are not apparent in a woman’s remaining pregnant and delivering after 37 weeks. For these reasons, the use of AWs for social considerations is less likely to be considered medically acceptable than in circumstances involving some clinical justification. In English law, patients are not empowered to demand any particular form of prenatal care^81^ (including presumably *ex utero* gestation), and simultaneously, doctors cannot be compelled to perform medical interventions in non-emergencies.^82^ It is unlikely, especially in communal health systems, that pregnant women would be routinely enabled to opt for *ex utero* gestation without medical cause. However, the possibility of such a demand for an alternative to natural gestation to allow women to avoid any (social) risks in order to reproduce is something we should take seriously.

This possibility raises new ethico-legal (and socio-political)^83^ issues. Should the AW be seen as, and used only as, a healthcare resource or should it be welcomed as a new reproductive choice? Hammond-Browning argues that although partial ectogenesis provides many benefits to women carrying a pregnancy, ‘foetal welfare’ and ‘uterine experience’^84^ must be taken into account. She argues, therefore, that an elective foetal transfer would only be justifiable in instances of dangerous pregnancy and/or where it can be ‘shown to be in the foetus’s best interest’.^85^ In contrast, Kendal argues that the AW must be utilised as a reproductive choice in order to afford those of female reproductive biology the potential for equal opportunity, both in terms of enabling them to reproduce with minimal physical risks and to evade what she terms the ‘social burdens’ that she claims inevitably result from pregnancy.^86^ If women could be enabled to exercise greater choice about what risks they are willing to assume in pregnancy without risking their foetuses, to what extent does the law empower them to opt for *partial ex utero* gestation? I now proceed to demonstrate that if all foetal extraction before 37 weeks requires legal justification, then it is unlikely that the current legal framework would permit women to end their pregnancies in order to opt for AWT for social reasons. This is because these circumstances fall outside of the AA 1967 and, potentially, the defence at common law. Moreover, women may not be able to opt for AWs at lower thresholds of maternal risk, because the AA 1967 is drafted to justify the ending of pregnancy only in instances of severe risk.

## IV. THE LEGAL PERMISSIBILITY OF PREGNANCY TERMINATION

The AA 1967 specifies that the law regarding termination is contained in sections 58 and 59 of the OAPA 1861. The actus reus of unlawfully procuring miscarriage is completed when an individual takes any unlawful steps to procure miscarriage (by any means).^87^ The requisite mens rea is that the object of that individual’s actions is the procurement of miscarriage.^88^ The offence is committed only in the absence of a defence. Defences are clearly listed in the AA 1967, which I will explore in detail later in this article. The offence of unlawfully procuring miscarriage is broad in its construction and lacks detail. The possibility of prematurely ending a pregnancy without it necessarily resulting in foetal death was not envisaged when the OAPA 1861 was first constructed. Acts of Parliament that have subsequently added and amended defences to the offence of procuring miscarriage (AA 1967, Human Fertilisation and Embryology Act 1990) were also drafted with the reasonable assumption that termination of pregnancy usually results in foetal death, and that other forms of termination later in pregnancy to save a pregnant woman’s life are ‘premature deliveries’ that only occur later in pregnancy, and in circumstances encompassed in the AA 1967. The offence of unlawfully procuring miscarriage, therefore, was deliberately designed to be all encompassing due to the limited knowledge of pregnancy and foetal development in 1861, and to include all the various different methods of procuring abortion that could be used by back-street abortionists.

The advent of AWs, and other technologies, that have enabled the possibility of prematurely ending a pregnancy without causing foetal death, is a blind spot for many areas of the law. The broad construction of the offence, in light of advancing technology, causes increasing uncertainty. This is primarily because the offence turns on the definition afforded to miscarriage,^89^ and subsequently how we might then define an ‘unlawful miscarriage’, as this is somewhat dependent on the meaning of miscarriage itself.

The term ‘abortion’ featured in the first British statute to regulate terminations, Lord Ellenborough’s Act of 1803,^90^ but was subsequently abandoned in other statutes until the AA 1967. The term ‘termination of pregnancy’ was not used in law until the AA 1967.^91^ Notably, despite the use of these terms in more modern statutory provisions, there were no amendments introduced to change, clarify, or supersede the terminology of miscarriage in the OAPA 1861. Whilst miscarriage might seem straightforward in meaning, there are two possible interpretations with different consequences for how the offence is understood and where it applies, that could be adopted. For the purposes of this article, these interpretations must be explored in the context of terminations later in pregnancy. Dickens noted that ‘one may allege that “miscarriage” is the wider expression covering a removal or discharge of the contents of the uterus before natural birth’, however he also posited that the phrase in its natural meaning should be accepted as a synonym of abortion (a deliberate intervention to end a pregnancy resulting in foetal death).^92^ There has been limited further exploration of this issue, because we tend to think of terminations not intended to result in foetal death as ‘premature deliveries’ accounted for under the AA 1967. In the context of contemporary and future medical developments, however, the uncertainty in the meaning of ‘miscarriage’ may become more problematic because of the possibility of ending pregnancy in favour of *ex utero* gestation if AWs did replace conventional NIC.

*Smeaton*,^93^ the only case in which a court has been required to consider the meaning of miscarriage in the OAPA 1861, concerned the legality of the morning-after pill. The Society for the Protection of Unborn Children alleged that contraceptives designed to ensure the prevention of a fertilised egg to implant in the uterus amounted to a ‘criminal miscarriage’. Munby J was unsympathetic, holding that ‘whatever it may or may not have meant in 1861 the word miscarriage today means the ending of an *established* pregnancy, and there is no established pregnancy prior to implantation…’.^94^ It was held that miscarriage was ‘the expulsion of the contents of a pregnant uterus’ at any point in pregnancy.^95^ This interpretation of miscarriage is too broad, because it makes no reference to the outcome of the miscarriage. If this interpretation were correct, unlawfully procured miscarriage would be any deliberate cessation of pregnancy in the absence of a defence.

There are grounds to believe an alternative definition of miscarriage might be adopted if the question were re-examined, particularly in the context of later term pregnancy. *Smeaton*^96^ focused on the beginning of pregnancy. The judgment emphasised that an instrument of procuring miscarriage had to have the capacity of ending an established pregnancy. Later in pregnancy, there is no issue regarding the established status of a pregnancy. Instead, the question is a much more explicit one. Does (unlawfully) procuring miscarriage necessarily involve intent to cause foetal death? There was a heavy reliance in the *Smeaton* judgment on medical evidence; many expert witnesses were called and medical texts were utilised. Munby J was clear that the definition of miscarriage that he arrived at was also ‘the current understanding of what is meant by miscarriage when used by lay people in the popular sense’.^97^ This implies that medical and lay opinion of what a miscarriage involves is relevant in understanding what it legally encompasses. When the OAPA 1861 was drafted there was no comprehension that there might be the possibility of ending a pregnancy without foetal death being both a necessary part of, and the intention of, the process. Common understandings of, and some medical definitions^98^ of, miscarriage include death as integral to the process. The second potential interpretation of miscarriage is, therefore, the ending of an established pregnancy resulting in foetal death. This will be referred to as ‘miscarriage as foetal death’. The difference between the two interpretations is that one is concerned with the condition of the foetus, and the other is not.

The actus reus and mens rea of the offence differ wildly depending on which interpretation is adopted. If miscarriage means any deliberate cessation of pregnancy (in the absence of a defence) then ending a pregnancy in order to facilitate *ex utero* gestation would fall within the parameters of procuring miscarriage (and, therefore, a doctor would need to demonstrate lawfulness in common law or under the AA 1967). However, if miscarriage necessarily includes only terminations that result in foetal death, ending a pregnancy to opt for *ex utero* gestation would not be a criminal matter so long as the intention was that the foetus survives the ending of pregnancy. There is an important distinction to be remembered here between endings to pregnancy where foetal death can be considered deliberate, and those where it can only be considered incidental.^99^ It is necessary that we ascertain which interpretation is, and should be, adopted in the criminal law. Medical technologies, of which AWs are just one, are increasingly blurring the distinction between those events that we would conceptualise as ‘medical premature deliveries’ and the deliberate unlawful ending of pregnancy. These technologies are making it more likely that questions surrounding the parameters of unlawfully procuring miscarriage will become a real issue in obstetric practice. Is ending a pregnancy to opt for an AW the unlawful procurement of miscarriage and thus in need of legal justification? If so, are there available defences in the AW context? This investigation, which I now undertake, involves examining the meaning of ‘miscarriage’ and the meaning of ‘unlawful’, as used in section 58. It should be noted that such an investigation of the law would not be necessary if termination were decriminalised, as questions of criminal liability would become irrelevant.

## V. DOES ALL ‘FOETAL EXTRACTION’ AMOUNT TO THE *UNLAWFUL* PROCUREMENT OF MISCARRIAGE?

In this section, I explore the possible interpretations that might be afforded to ‘miscarriage’ and ‘unlawful miscarriage’ in the OAPA 1861. I consider whether the law could be interpreted to mean that all deliberate endings to a pregnancy are *prima facie* criminal, or whether only terminations resulting in foetal death are ‘miscarriages’ for the purposes of the criminal law. I consider the possible arguments that might be offered to support each interpretation and I analyse the law from both of these differing perspectives to show that there would be different conclusions reached about the legal consequences of foetal death depending on the analytical lens adopted. I demonstrate that we cannot be certain how miscarriage would be interpreted if the matter came before a court of law, as there are compelling reasons that mean both are plausible accounts of the law. However, I advocate that (assuming criminalisation is appropriate), the most ethically sound operation of the law is dependent on unlawful procurement of miscarriage being understood as referring only to those terminations intending to cause foetal death. I argue that law reform is necessary.

### A. Are *all* Deliberate Endings to Pregnancy Presumptively Criminal?

By looking at other statutes concerning the law of terminating pregnancy, it could be argued that Parliament did not intend that miscarriage would refer only to deliberate interferences to end a pregnancy that is intended to result in foetal death. There is significant overlap between the offence of child destruction contained in the Infant Life (Preservation) Act 1929 (ILPA 1929)^100^ and the offence of procuring miscarriage in the OAPA 1861. Terminating a pregnancy that results in the death of a foetus ‘capable of being born alive’^101^ would amount to an offence under both provisions.^102^ Grubb posits that the only distinction between the two statutes is that the offence of procuring miscarriage can be committed before the 24-week viability threshold.^103^ There is, however, a further distinction Grubb did not account for. The ILPA 1929 is explicit that the actus reus of child destruction is complete only when action results in foetal death, whereas the OAPA 1861 is too vague to be explicit to this effect. Brazier and Harris posit that the language used to construct the OAPA 1861 and AA 1967 imply that ending pregnancy and foetal death are ‘*one and the same*’.^104^ Alghrani and Brazier expand on this, arguing that ‘s.58 envisages [and criminalises] a process inevitably designed to kill the foetus’.^105^ The offence does imply that there is usually some action in procuring a miscarriage that is associated with the causing of death. The provision makes references to some methods of procuring miscarriage that might cause damage to a foetus: ‘any poison or other noxious things’.^106^ The offence is not explicit, however, that only those methods of procuring miscarriage that would have noxious effect, are criminal. It could be argued that the offence is specific to the contrary because it states that ‘any other method whatsoever’^107^ of ending a pregnancy is criminal. Any other method could have been a direct reference to action that was not in any way designed to cause immediate harm to the foetus. It certainly encompasses that possibility in its plain meaning.

Moreover, the offence of unlawfully procuring miscarriage was constructed as an offence committed against the pregnant woman’s body, not against an unborn foetus. The offence first appears in the OAPA 1861, and the law has always been clear, even in 1861,^108^ that the foetus is not a person in law.^109^ As already noted, where it is a person other than the pregnant woman accused of unlawfully procuring miscarriage, there is no requirement that the woman is actually pregnant when an attempt is made to procure miscarriage.^110^ This suggests that the condition of the foetus is not the primary concern of the offence, because there need not be any foetus at all for the offence to be committed. In either case, no reference is made at all to the foetal body, its condition, or its experience. It seems, therefore, almost counter-intuitive, to read any meaning into ‘miscarriage’ that involves the condition of the foetus.

Finally, the AA 1967 provides a defence to terminations of pregnancy on a medical model in limited instances. It is interesting that defences were provided expressly to cover those cases that would conventionally be termed ‘premature deliveries’: the ending of a (later term) pregnancy to save a woman’s life with attempts to ‘save’ the preterm neonate pre-empted and engaged. The fact that explicit legal authority for such actions was deemed to be necessary in the form of a defence in the AA 1967 (to the extent that implicit common law defences were codified for this context), strongly implies that the ending of the pregnancy is always *prima facie* criminally relevant, because even those instances where there is an attempt to save the foetus are encompassed in the AA 1967.

### B. Is it Only Terminations Intended to Cause Foetal Death that are Criminal?

There are, however, also compelling reasons to suggest that miscarriage would be interpreted more narrowly. Criminal statutes are generally interpreted narrowly, in favour of an accused person,^111^ as part of the principle of fair warning.^112^ It is unlikely that when first drafted Parliament intended the offence to cover instances where individuals had not attempted to undertake a termination resulting in foetal death (irrespective of whether this was the result). This is because there was no such thing as *induced* premature delivery in 1861. Knowledge of obstetric complications was limited. Deliveries could not be chemically induced.^113^ Caesareans, while possible, were not yet routinely available or safe (and mortality rates were high).^114^ There was no NIC to support premature babies.^115^ Premature labours were only spontaneous occurrences^116^ dangerous for the pregnant woman and foetus. These factors combined meant doctors had no incentives to attempt ‘premature deliveries’. It could be argued, therefore, that since Parliament could not have intended to render these circumstances the concern of the criminal law, miscarriage should not be interpreted to have this effect.

There are clues in language elsewhere in the offence that might be used to argue that only conduct intended to cause foetal death should be considered criminal. It is not to be ignored, as Alghrani and Brazier, and Brazier and Harris suggest (discussed above), that Parliament specified only the language of ‘violence’ in the offence. The fact that non-violent methods of ending a pregnancy were not explicitly excluded does not mean the offence meant to encompass those situations. The non-exclusion of ‘non-violent’ means of termination may have intended to refer to scenarios in which the foetus did not survive because it was removed unharmed from the uterus before the end of gestation and, being underdeveloped, it died ‘naturally’ *ex utero*. Such arguments, however, are likely to be insufficient without creative judicial interpretation. Principles of statutory interpretation could direct us to the conclusion that the term miscarriage encompasses only the deliberate ending of pregnancy intended to result in foetal death. *Smeaton* was an instance in which the law was faced with an unanticipated medical development.^117^ In 1861, contraception as efficient as the morning-after pill would have been equally as unimaginable as partial ectogenesis. Parliament was not intending when legislating on abortion ‘to ban abortion whilst permitting contraception. It was simply legislating to punish abortion’.^118^ The same is true in this instance; Parliament was not attempting to ban ‘premature deliveries’. It was making rules about abortion. How should a term used in ignorance of future medical developments be interpreted? In *Smeaton*, Munby J approached the issue using the principle of updating construction.^119^ The language, and the parameters of the language, used in the 1861 Act cannot be abandoned because this is how Parliament chose to legislate,^120^ and thus the question of interpreting the boundaries of the offence of procuring miscarriage in the context of AWs must be determined using the given language. This does not mean, however, that the language used in the OAPA 1861 should convey only the meaning it had at the time it was written.

In *Smeaton*, Munby J held that the 1861 Act was an ‘always speaking Act’ and there was significance in Parliament having left the word ‘miscarriage’, so central to the offence, undefined. There have been plenty of opportunities even following this judgment for Parliament to intervene and yet a definition has not materialised. Accordingly, Munby J observed that miscarriage should be ‘interpreted as it would be currently understood and it should be interpreted in light of the best scientific and medical knowledge that is available to the court’.^121^ In advancing this conclusion, Munby J was relying on the approach adopted by the House of Lords in *R v Ireland*.^122^ Lord Steyn was explicit that in interpretation exercises ‘courts of law must act on the best medical insight of the day’.^123^ The judiciary are likely, and entitled, to use medical evidence and medical definitions as an external aid to interpreting^124^ the OAPA 1861. This was an important exercise in the *Smeaton* judgment, in which expert advice was sought as to the meaning of an established pregnancy that could be ‘miscarried’. The same approach is likely to be taken to determining the modern meaning of miscarriage and whether this is intended to refer only to endings of pregnancy intended to result in foetal death. There has consistently been deference to medical opinion on the part of the judiciary in the realm of abortion law.^125^

There are varying medical definitions of miscarriage. There is no uniformity in definitions in explicitly referring to foetal death. Miscarriage is often defined in medical dictionaries as ‘the spontaneous loss of pregnancy before 24 weeks’.^126^ Such definitions are confusing because they refer to the loss of a pregnancy as opposed to the deliberate ending of a pregnancy. They are still, however, helpful in demonstrating that the term miscarriage is most often used in situations in which foetal death has occurred or is inevitable. Other medical dictionaries define miscarriage as ‘the loss of the foetus’,^127^ more explicitly indicating that foetal death is the consequence of a miscarriage. Others, however, vaguely refer to miscarriage as only the ‘induced expulsion of a human fetus’.^128^ Medical opinion would be likely, on balance, to support the opinion that procurement of miscarriage encompasses only endings to a pregnancy that result in foetal death. Medical books mentioned in the *Smeaton* judgment were implicit that this was the case, and even defined a spontaneous miscarriage earlier in gestation in juxtaposition to a spontaneous premature labour later in gestation.^129^ Moreover, management of difficult complications in pregnancy by its premature ending is common professional practice. The methods undertaken to end a pregnancy are often intended to ensure the foetus survives delivery. In the chosen methods of termination, the juxtaposition between induced loss of pregnancy resulting in foetal death and induced delivery of pregnancy is easy to see.

In interpreting the relevant provisions, beyond only taking a literal approach examining language, the judiciary are empowered to interpret terms in light of what their use in the provision was attempting to achieve.^130^ The two ‘purposes of the … [OAPA 1861] were plainly the protection of women and the protection of the unborn’.^131^ The Act intended primarily to limit abortion, and to protect women from the dangers of termination (inevitable in 1861 because even terminations performed by doctors were unsafe) and, in particular, from back-street abortion. The danger posed by all premature endings of pregnancies is now minimal in medical settings, and the concern regarding back-street abortion almost eliminated. It is believed that there has not been a death resulting from back-street abortion in England and Wales since 1982.^132^ Thus, in placing limitations on access, the OAPA 1861 serves the purpose of enforcing some protection for foetuses. This raises the important issue, however, of how far that protection was intended to extend. This issue of quantifying protection was explicitly acknowledged but dismissed as irrelevant to the issue before the court in *Smeaton*.^133^ Munby J asked ‘how far back [in pregnancy] does the protection afforded by the Act extend?’^134^ In the case of ending a pregnancy in order to facilitate gestation *ex utero*, we need to be asking a similar question about what the protection afforded to the foetus in the law substantively entails. Specifically, how late in pregnancy is this protection extended? I argue that unlawful procurement of miscarriage *should* be interpreted to refer only to those incidents intending to cause foetal death, however, this is by no means a concretely established interpretation of the law; there are equally compelling reasons to believe that the law as it is currently written necessitates that, legally, miscarriage encompasses any deliberate cessation of pregnancy. Reform of the law for clarity (provided decriminalisation is not possible),^135^ is necessary.

If the legal definition of ‘miscarriage’ necessarily included foetal death, this would result in the most ethically sound operation of the law (again, making the questionable assumption that criminalisation is appropriate)^136^ because it would not require doctors, acting on behalf of women, to justify the ending of a pregnancy in favour of *ex utero* gestation. Intuitively, if we accept the legitimacy of a criminal, and medical, model of termination provision, there is a meaningful distinction between procurement of miscarriages later in pregnancy that should be considered criminal, and thus in need of some justification,^137^ and endings to pregnancy that are in need of no such explanation. That distinction would be the intention of the doctor in bringing the pregnancy to an end. In reality, when it is decided that a later term pregnancy should be ended, what efforts are made to preserve the foetus are influenced by viability, and determined by the approach and subsequent actions adopted by attending doctors, influenced by the wishes of the parent(s). Grubb observes that often, ‘providing it is not inconsistent with saving the mother, saving the foetus and producing a live birth will be desired by the mother. Therefore, these abortions amount to induced labour… A late abortion in these circumstances is consistent with foetal survival’.^138^ If decisions regarding mode and timing of ending pregnancy are made with the intention that a foetus will survive, this seems like a ‘premature delivery’. This ending of a pregnancy would be hard to criticise as ‘unethical’ because it encompasses action being taken to preserve a woman’s life and health, while simultaneously respecting their desire to protect their foetus. With the direction of the pregnant woman, thought is being directed to the foetus, and action can be taken to promote its survival. A ‘premature delivery’, in an intuitive sense, implies an intention to ‘birth’ the developing human entity to the world. The term ‘delivery’ implies the conveyance of a good and the connotation is that the usual outcome of pregnancy (a baby) would be that good.

Conversely, miscarriage implies that the conveyance of the usual good of pregnancy (the baby) has failed or is intentionally prevented. If decisions regarding mode and timing of ending pregnancy are made with no intention towards the survival of, or actively to secure the death of, the foetus, this seems, intuitively, the kind of miscarriage the criminal law was intending to regulate. The distinction is not based on when an attempt is made to end pregnancy, making assumptions about likely outcomes, but on a combination of why, how, and when the deliberate intervention into pregnancy is undertaken. This distinction is easily read into the law if, first, miscarriage is defined as the termination of pregnancy resulting in foetal death (thereby requiring a mens rea of intent to cause foetal death), and/or, secondly, if the word ‘unlawful’ in section 58 were, by definition, to exclude those instances in which there was no foetal death. Making the mens rea the distinction—considering the object of the ending of the pregnancy and/or intentions of the person ending the pregnancy—provides a consistent approach to determining what endings to a pregnancy are ‘in need of justification’ that would match common intuitions about the purpose of the criminal law. Moreover, considering intention is future-proof, in that it could continue to isolate activities for regulation, even with the introduction of technologies like AWT.

It is possible that I have overestimated the extent to which Parliament was, in legislating to prohibit procurement of miscarriage, concerned with the protection of foetal welfare. It is certainly plausible that the OAPA 1861 was intended to be broadly interpreted and is interested in reinforcing the heteronormative regulation of female bodies. If miscarriage is interpreted broadly to mean any unlawful deliberate cessation of pregnancy, regardless of outcome, this effectively prioritises the protection of foetuses over the interests of women (and thus does not protect women). This is because it places a burden on women to provide some legal justification for their decision to opt for artificial gestation. The criminalisation of opting for *ex utero* gestation would have very negative, emotional, and moralistic connotations, such that, even if it was *prima facie* defensible, it would have real consequences for women. The construction of ‘criminal abortion’ in the law is used to implicate activities deemed to be ‘deviant’.^139^ Requiring some justification to avoid criminal sanction, even if it is easily done, still has the impact of stigmatising that decision, categorising it as ‘in need of explanation’, and labelling women who want to escape some of the burdens of pregnancy as making a *prima facie* ‘bad’ choice.

Moreover, if all endings to a pregnancy were unlawful outside of the applicable defences in the AA 1967,^140^ this could place a legal obligation to undertake gestational work (remaining pregnant) in place of alternative forms of gestation on pregnant women who are not facing an immediate serious risk to health or any risk to life.^141^ Was the Act intended to place such an onerous requirement on women and entirely subjugate their right to bodily autonomy and integrity to privilege foetuses? In 1861, the answer would have been ‘yes’ because ending a pregnancy was unlawful in any circumstances, save where necessary to save a pregnant woman’s life.^142^ However, would Parliament have intended to place this onerous obligation on women in a world in which AWs were available to free women of pregnancy without corresponding foetal death? In interpreting the meaning of miscarriage, the impact of an all-encompassing definition of termination on the rights of women should be considered. Bodily autonomy is afforded the highest respect in law.^143^ Today, interference with women’s choices about their body regarding remaining pregnant is only arguably justifiable because of the state’s interest in the foetus.^144^ There are good reasons to believe this is not the case, but for the purposes of the current discussion this is assumed. If gestation can be continued without the use of a woman’s body, there should be no legal requirement that she remain pregnant for any reason.^145^ If miscarriage encompasses only termination resulting in foetal death, the state is still able to enforce its ‘interest’ in foetuses. The OAPA 1861 would penalise only individuals who attempt to procure termination resulting in foetal death without an applicable defence. This aspect of the criminal law would not concern itself with attempts to extract foetuses for continued gestation *ex utero*. For this reason alone, if criminalisation is thought appropriate, ‘miscarriage as foetal death’ should be adopted as the definition of miscarriage in criminal law.

Another important point to be made about the parameters of the offence is the use of the term ‘unlawful’, further qualifying the meaning of miscarriage in the 1861 Act. In *R v Bourne,^146^* Macnaghten J held that the explicit use of the term ‘unlawful’ in the offence of procuring a miscarriage was not ‘meaningless,’ and thus imported the defence that had always been in the common law. According to this judgment, a doctor ending pregnancy in good faith and to preserve a pregnant woman’s life or health does not act unlawfully.^147^ Whilst this can be conceptualised as a defence, and one that is now incorporated into the AA 1967,^148^ this case demonstrates that the use of the term ‘unlawful’ in the construction of the offence is thought to be crucial. The fact that the word features no less than four times in section 58 means it is central to the actus reus of ‘unlawful miscarriage’. It is arguable that foetal extraction with the intent of completing gestation would not be considered unlawful and, in not satisfying an actus reus requirement, there would be no need to consider defences. The 1909 *Re McCready* judgment^149^ in the Supreme Court of Saskatchewan (Canada) came to such a conclusion. The Canadian Criminal Code (at that time) closely mirrored the provision in the 1861 Act, specifying that the offence was committed when the action to procure miscarriage undertaken with intent to procure miscarriage was unlawful.^150^ Lemont J held that, on the facts, it was not possible to confirm that the miscarriage was not necessary to preserve the woman’s life, ‘in which case it is not unlawful’.^151^ He posited that ‘every miscarriage brought on by a physician is not unlawful …’^152^ and, despite the evidence that the defendant was involved in the procurement of miscarriage, he found no reason to believe it was an unlawful one and, therefore, discharged the defendant.^153^ This case strongly suggests that the actus reus of the offence centres on *unlawful* miscarriage.

The *Bourne*^154^ judgment is also good law supporting the same conclusion in the English context. Just as it was held in this case that saving the life of, or preserving the mental health of, the pregnant women was lawful under section 58, it might be that ending a pregnancy whilst preserving the foetus would also be a ‘lawful miscarriage’. As Dickens points out, however, ‘one may question historically whether the word was intended or realised by the legislature to imply what Macnaghten J saw in it’.^155^ The significant legal developments since *Bourne*—the passing of the AA 1967 and subsequent parliamentary debate about those circumstances in which the ending of pregnancies is thought to be lawful—raise the question of whether a different approach would be taken to deny that such importance be placed on ‘unlawful’ in circumstances not proscribed by that Act. It remains to be seen how this turn of phrase would be interpreted, and whether there would be any need to look to the AA 1967. For the sake of thoroughness, it is important that we consider the circumstances in which there would be a defence to unlawfully procuring miscarriage if *all* deliberate endings of pregnancy (including those instances where the intention is to continue gestation *ex utero* for medical or social reasons) were *prima facie* criminal. If a defence can be successfully raised in situations where a doctor terminates a pregnancy intending that the foetus will continue gestation *ex utero*, then their conduct would not be unlawful.

## VI. ARE THERE AVAILABLE DEFENCES TO *UNLAWFULLY* PROCURING MISCARRIAGE?

For all terminations before 24 weeks’ gestation, there is a defence available to medical practitioners under the AA 1967.^156^ Access to conventional terminations of pregnancy is relatively liberal before 24 weeks because section 1 (1) (a) of the AA 1967, the defence under which the majority of abortions are performed,^157^ is so broad that it renders ‘every pregnancy legally terminable within the first 24 weeks’.^158^ In practice, doctors do not tend to justify their decision to perform termination before 24 weeks on clinical grounds. However, this does not mean that it is necessarily lawful for doctors to provide terminations (even to allow for *ex utero* gestation) before 24 weeks for any non-medical or social reason. This is because the so-called ‘social ground’ for abortion is *still* framed in medical terms; it provides a defence before 24 weeks where the risk of continuing the pregnancy is *greater* than the risk of ending pregnancy. Methods of conventional abortion have now developed to the point that it is seemingly statistically always the case that performing an abortion (usually drug induced) within the time frame that most are requested is less risky than continuing pregnancy.^159^ It seems unlikely that the foetal extraction procedure necessary for transferal to an AW would be less risky for a pregnant woman than remaining pregnant (or having a conventional termination) in the vast majority of circumstances. Of course, all pregnancy is inherently risky, but if the process of foetal transfer were to involve an intricate caesarean procedure,^160^ it might be argued that this is far riskier than continuing the pregnancy if the pregnant woman is healthy and a natural delivery is anticipated. Moreover, this provision of the AA 1967 does not mean that all women are legally entitled to a termination of pregnancy (by any method, with any outcome). The AA 1967 has effectively ‘transferred’ the right to self-determination, specifically regarding decisions about whether to remain pregnant, from women to the medical profession.^161^ The way that the law is framed means women have no positive right to end their pregnancy. Women must convince a doctor that they require intervention into their pregnancy, and that they are entitled to it because their circumstances fit within the medical model.

Furthermore even where it may be lawful for doctors to perform interventions to end a pregnancy, they are under no obligation to do so unless it is an emergency.^162^ As already noted, the law is clear that patients are not entitled to demand any particular treatment, and doctors cannot be compelled to provide treatments they are uncomfortable with.^163^ Women are effectively subjected to an enormous constraint on their decision-making, because they are empowered only to make decisions about ending pregnancies for reasons that satisfy medical practitioners. This is even assuming that medical practitioners would be willing to end pregnancies in favour of *ex utero* gestation before 24 weeks for less urgent medical considerations, or non-medical reasons. This raises some ethical issues for exploration about the role of medicine in reproduction, the parameters of what treatment is, and when it can be demanded. This discussion is important to revisit, but is beyond the scope of this article.

For women experiencing high-risk or dangerous pregnancies, it would potentially be lawful for them to end their pregnancies in favour of *ex utero* gestation before 24 weeks gestation. After 24 weeks, a lawful defence to a deliberate ending of a pregnancy becomes much harder to establish. The two relevant grounds of the AA 1967 that could be invoked as a defence to the circumstances of potentially dangerous/high-risk pregnancies are risk to the pregnant woman’s life^164^ and risk of grave, permanent injury to the physical or mental health of the pregnant woman.^165^ These defences are available when two medical practitioners form the opinion,^166^ in good faith, that a pregnant woman’s circumstances fall within these grounds. There is no time limit imposed on these grounds for termination. They are, therefore, harder to establish and require ‘clear proof of the more serious danger specified’.^167^ The separation of these two grounds related to the pregnant woman’s condition in the statute, which distinguishes life from health, is an attempt to demark that the ground of risk to *health* requires more substantial proof than risk to life.^168^ In the following sub-sections, I consider what level of risk is sufficient to justify deliberately ending pregnancy, and whether these legal requirements satisfactorily account for the change in risk perception that might follow AWs.

### A. Risk to Life

Doctors need not wait until a pregnant woman is in peril of immediate death to end a pregnancy^169^ since, as noted, there is a defence to unlawfully procuring miscarriage when pregnancy poses a risk to the pregnant woman’s life that is greater than the risk of termination.^170^ The risk to life and risks of termination must be weighed against each other.^171^ Most premature deliveries that are currently undertaken are attempted because the pregnancy poses specific risks to the pregnant woman’s life.^172^ The method by which the pregnancy is ended often carries the same risks as delivery at the end of the normal gestational period when a pregnancy has to be managed.^173^ The risk of termination will have to be assumed at some point in order for the pregnancy to end, whether before, or at the end of, the normal period of pregnancy. Therefore, when there is risk, it is often easy to demonstrate that the risk of remaining pregnant is greater than the risk of inducing the end of the pregnancy by vaginal expulsion or caesarean. This defence encompasses the decision-making calculus currently adopted in obstetric practice.^174^ However, if AWs shift perceptions about which pregnancies are risky, what does this mean for the interpretation of ‘risk to life?’

The explicit balancing exercise that the defence requires means a lower threshold of risk to life is not easily read into the provision. Even if a doctor believes a lower level of risk earlier in a pregnancy justifies ending the pregnancy (because they no longer concern themselves with balancing risk against the impact of foetal immaturity), it will not necessarily mean they can form the opinion in good faith that ending the pregnancy carries a lesser risk *to life* than remaining pregnant. The serious conditions that termination is necessary to manage at present will fit within the defence in milder forms and perhaps earlier in their onset. Conditions that do, or will, pose some risk to life, can be balanced against the risks of the method of ending the pregnancy accordingly.

AWs, however, could result in pregnant women with a broader range of medical complications, some of lesser severity, wanting to end their pregnancies because there is a better guarantee that this will not result in foetal loss. Consider a pregnant woman struggling to manage unrelenting nausea. Her symptoms may not pose an obvious risk to life,^175^ and would certainly not constitute such a risk that continuing pregnancy could be described, in good faith, as more of a threat than the risk of complications in a medically induced ending of pregnancy. This is particularly evident in circumstances where a routine vaginal birth at the end of the normal gestational period is anticipated. The question is ultimately whether a doctor could, in good faith, come to the opinion that her patient’s circumstances are encompassed under the defence.^176^ It is notoriously difficult to prove a doctor did not form their opinion in good faith.^177^ There seems to be scope, however, to question the opinion of a doctor who believes sickness, swelling limbs or limited mobility during pregnancy is a threat to life, or a greater threat than induction or caesarean. It appears that a ‘foetal extraction’ for gestation *ex utero* in an AW before the end of the usual gestational period would only be lawful by virtue of section 1 (1) (c) of the AA 1967^178^ outside of conventionally serious circumstances in which the threat to the woman’s life is easily observable.

### B. Risk of Grave, Permanent Injury to Physical or Mental Health

For a doctor to avail herself of the defence related to preventing a grave and permanent injury to a pregnant woman’s physical or mental health, she must believe that the termination is necessary to prevent the injury, but it need not be immediately necessary.^179^ The doctor can act where permanent and grave injury is foreseen.^180^ There is no further statutory guidance as to the meaning of ‘grave’ and ‘permanent’ and what conditions would be severe enough to establish this defence. This is open to the interpretation of doctors forming their opinion in good faith, though it is clear that only serious and long-term illnesses resulting directly from the continuation of pregnancy would be sufficient.^181^ There is no balancing exercise; the defence is not a question of whether there is a risk of injury from remaining pregnant greater than the risks associated with early ending of pregnancy.^182^ The doctor must be of the opinion that grave, permanent injury is ‘reasonably certain to occur if pregnancy [is] continued’.^183^ This reinforces the notion that the defence is extended to serious injury only.

In the 1990 House of Lords debates about amendments to the AA 1967, the Lord Chancellor stipulated that the terms ‘grave’ and ‘permanent’ were deliberately used to create a stiff legal test. He suggested examples of sufficiently serious injury included ‘where [the pregnant woman] has severe hypertension and continuation of the pregnancy might result in permanent kidney, brain or possibly heart damage’.^184^ Morgan and Lee suggest further examples; ‘mild preeclampsia; breast or cervical cancer…. uncontrolled diabetes; [and] conditions which may improve or deteriorate during pregnancy, such as asthma or epilepsy…’.^185^ It is interesting that the debate focused on women’s physical, rather than mental, health. This defence was clearly designed to encompass the kinds of decisions currently made in practice. In the House of Lords, there was implicit acknowledgement that the defence was designed with ‘premature deliveries,’ rather than terminations resulting in foetal death, in mind. There was explicit reference made to the bond that develops between woman and foetus throughout pregnancy,^186^ and Lord Mackay remarked that later in pregnancy he expected that ‘the method of termination would be selected in the best interests of the woman, but the intention would be to deliver a living baby where possible’.^187^ It was anticipated that terminations necessarily resulting in foetal death would usually only be carried out when this was the only possible method of ending pregnancy that would spare or significantly reduce the likelihood of injury to the pregnant woman. An example would be those instances where there is a birth canal obstruction that requires cranial crushing of the foetus in order to extract it.^188^ The AA 1967, however, still provides a defence to unlawfully procuring miscarriage when the pregnancy threatens serious injury and a miscarriage resulting in foetal death is chosen for any reason.^189^ In practice, it is most often the case that when pregnancies are ended early by interference, the pregnant woman wants her foetus to have the best chance of surviving and decisions are made regarding the timing and method of delivery accordingly.

If the decision-making calculus regarding ending pregnancies starts to shift because of AWs, there may be instances when the ending of a pregnancy might be medically advantageous to a woman (given the reliable alternative form of gestation), but where pregnancy is not anticipated to cause a ‘grave permanent injury’ to her health. The serious conditions for which intervention to end pregnancy is currently recommended, even earlier in their onset, would still be encompassed under this defence. There are compelling arguments that all pregnancies cause grave and permanent injury to women’s bodies.^190^ However, the defence is constructed in such a way as to prevent such an argument being legally entertained.

Many women’s experiences of comparatively milder complications or ‘side effects’ during gestation make their pregnancies difficult. For some women, it would be better for their physical and mental health not to experience these symptoms. However, these side effects are unlikely to be considered ‘grave,’ a threat to long-term health, or likely to cause serious and/or long-term damage to health. Morning sickness, limited mobility and swollen limbs are temporary hindrances to health that will end with the pregnancy; therefore, they could not be described as long-term injuries. There will be a huge difference of opinion as to how grave these symptoms are to experience. Many women who desire to remain pregnant *despite these side effects* do so because they want a future child, not because they enjoy pregnancy. If AWs were available as a reliable alternative to pregnancy, continuing gestation and better guaranteeing the desired outcome, it is plausible that some women would want to end their pregnancies, opting for AWs. Continuing a pregnancy that encompasses difficult symptoms that pose a hindrance to short-term health or quality of life would not be in these women’s interests. However, the AA 1967 provides no express permission for a doctor to procure the ending of a pregnancy to improve a woman’s short-term heath, or (what might be perceived as), quality of life after 24 weeks.

Notably, this ground of the AA 1967 also provides a defence if a doctor believes that termination would prevent grave and permanent injury to a woman’s *mental* health. In *Bourne*, the judge stressed that termination would be lawful if a doctor was of the reasonable opinion ‘that the probable consequence of the continuance of pregnancy would be to make the woman a physical or mental wreck’.^191^ In this judgment, recognition was afforded to the inevitable emotional and psychological trauma if a young girl was forced to carry a foetus conceived by violent rape. Quite what other circumstances would be considered legally sufficient here, or under the AA 1967, is uncertain.

In short, without amendment to the AA 1967, there is mismatch between the law and the decision-making calculus based on AWs that obstetricians would want to deploy in women’s interests. Current statutory provisions potentially require women to sustain their foetuses by remaining pregnant, rather than opting for artificial gestation. This framing of the law, even if only symbolically, subjugates the female body for the purposes of reproduction. This seems *particularly* callous if the female body is not necessary for gestation. This is an ethical issue deserving of more attention in the legal discourse surrounding AWs.

### C. Doctors’ Discretion?

The defences in the AA 1967 can be interpreted broadly. The legality of ending pregnancy is dependent on whether doctors acting in good faith *perceive* that a pregnant woman’s circumstances meet the grounds in the AA 1967, not on whether their circumstances actually do.^192^ Furthermore, the defences in the AA 1967 were not the first available for doctors procuring miscarriage. The common law implicitly provided doctors with a defence to unlawful procurement of miscarriage long before 1967. In 1938, *R v Bourne*^193^ highlighted that doctors could lawfully end pregnancies where their object was to save the life of the mother or prevent permanent damage to their health.^194^ This case demonstrates that the use of the term ‘unlawful’ in the construction of the offence of procuring miscarriage means that there is significant judicial discretion in determining which procurements of miscarriage are ‘unlawful’. There are several reasons, therefore, to believe that the power to determine the legality of procuring miscarriages has been placed firmly in the hands of the medical profession. It seems unlikely that any decision-making process regarding ending pregnancy that is not intended to harm the pregnant woman or foetus, therefore, would be treated as criminal.

First, judges are likely to direct juries in such a way that encourages acquittal on charges of procurement of miscarriage where a doctor did not cause foetal death, or intend for miscarriage to result in foetal death. On these charges in the past, judges (even before public opinion about abortion liberalised, and in circumstances where termination inevitably would result in foetal death) have been keen to emphasise the difference between a medicalised ending of pregnancy and the illegal actions of ‘back-street abortionists’. In *Bourne,^195^* MacNaughten J emphasised on three separate occasions in his jury direction how the actions of the doctor procuring a miscarriage for a woman following his perceived belief that it was in her interests was different to what is normally understood as ‘illegal abortion’.^196^ A similar emphasis might be attached to a medical decision to procure miscarriage based on a decision-making calculus with medical merit, even if focused on a lower level of risk.

Secondly, a jury is unlikely to convict a doctor^197^ unless there is evidence that the doctor was behaving with blatant disregard for the purpose of the law. If the intention of a doctor in ending a pregnancy was not to cause foetal death and to best protect the pregnant woman’s health, it is hard to imagine any jury questioning the decision-making process unless things go wrong and/or the doctor is shown to be acting in bad faith. Thirdly, judges are likely to quash convictions that they feel may be unsafe. In the *Paton*^198^ judgment, Baker P was explicit that a judge who sought to interfere with the discretion of doctors under the AA 1967 would be both bold and foolish, *‘*unless, possibly, where there is clear bad faith and an obvious attempt to perpetrate a criminal offence’.^199^ It is unlikely that a doctor who sought to preserve the life of both the pregnant woman and her foetus would be seen as making an obvious attempt to behave criminally. Even before the advent of AWs, and before a doctor undertaking an earlier termination could claim they explicitly intended that the foetus would continue gestation *ex utero*, the judiciary have been unwilling to question doctors decision-making in this arena. In *R v Smith (John)*^200^ the Court of Appeal, overturning a doctor’s conviction of criminally procuring miscarriage, posited that without clear evidence that behaviour was not within the reasonable bounds of professional practice and medical probabilities, ‘a verdict against a doctor is often unlikely to be unsafe’.^201^ And Keown has argued that ‘medical control of pregnancy and childbirth has long been established as has the termination of pregnancies’.^202^

It is thus unlikely that, whatever the legal definition of miscarriage, there will be a charge or a conviction for unlawful procurement of miscarriage upheld against a doctor who ends a pregnancy intending that gestation is completed *ex utero*. Despite this, the legal uncertainty that I have examined is problematic, because ambiguity violates the human rights of those subject to the law.^203^ Legal uncertainty has the potential to foster moral uncertainty amongst the medical profession,^204^ and potentially restrict the use of future medical technologies that might help reduce the physical burdens placed on women during pregnancy. It has long been observed that the AA 1967 firmly placed decisions about ending pregnancies into the ambit of the medical profession; however, as more avenues of potential choice open to women, it is important that the degree of medical control over those choices is re-examined. This degree of medical control over women’s decisions to end their pregnancy in favour of *ex utero* gestation is not justifiable for the reasons reflected in the termination jurisprudence thus far. This reasoning has focused on the ‘state interest’ in preserving a foetus’s limited right to be gestated and the state’s interest in life, and this does not apply in these circumstances. Women should be permitted to decide how long they wish to be pregnant. In this article, I have considered the case of dangerous pregnancies; however, some of my arguments may equally apply to those instances in which women want to opt out of undertaking gestational work in favour of an AW in a broader range of circumstances. In considering whether the law should interfere with a choice to gestate *ex utero*, there can be no objections from those who believe that foetal welfare should prevent (conventional) termination because foetal death is not at issue here. The fact that women are unlikely to gain greater control over their pregnancies even if/when future technologies enable greater choice, is an important problem that should be addressed. Sheldon observes that the medicalisation of termination decisions in the AA 1967 was useful to aid the partial de-politicisation of abortion, and to liberalise access to terminations for women in England and Wales. However, the framing of the AA 1967 has limited utility in ‘defending and furthering women’s reproductive rights…’.^205^ There are two reasons for this: first, because women both have no formal legal right to demand termination and, secondly, because ‘clinically unsupported restrictions on abortion provision have been retained as part of a system of tight medicalised control’.^206^ It is time to decriminalise all methods of pregnancy termination regardless of their intended outcome.^207^ In other words, it is time that we trust women to make pregnancy termination decisions.

## VII. CONCLUSION

In this article, I have considered the claim that AWT could relieve some of the physical burdens placed on women during pregnancy. I argued that AWT, with the capability to continue gestation, has the capacity to empower women to escape potentially dangerous pregnancies without risking their wanted foetuses (if this is their concern). Given the capacity of AWT to shift the focus of decision-making in obstetrics to empower women, it is important to consider whether those choices would be accessible to women under current legal provisions. The legality of ending pregnancy in English law still turns on the offence of ‘unlawfully procuring miscarriage’, to which doctors have a defence if they can demonstrate that the termination they provide is ‘lawful’ by virtue of the circumstances contained in the AA 1967, or potentially the more implicit necessity defence from *Bourne*.^208^ How the present legality of ending pregnancy to opt for gestation *ex utero* is determined depends on whether ‘miscarriage’ and specifically ‘unlawful miscarriage’, neither of which are usefully defined in any statute or case law, means the deliberate cessation of any pregnancy, or only those attempts to end a pregnancy in such a way that results in foetal death. I have demonstrated that there is ambiguity here; whilst an interpretation of ‘miscarriage’ as only encompassing those instances where there is intent to cause foetal death is the most ethically justifiable (if we accept that criminalisation is appropriate), such an interpretation involves judicial creativity.

It is important to determine what miscarriage means, because if miscarriage includes ending pregnancy to opt for *ex utero* gestation, this action would be *prima facie* criminal unless ‘lawful’ or unless a doctor could raise a defence. I demonstrated that it was unlikely that a doctor would be found guilty of unlawfully procuring miscarriage in instances where there was no attempt to procure foetal death in the process. However, women’s access to a choice to end pregnancy in favour of an AW could be based entirely on medical perceptions of risk. A question for further consideration remains: is it appropriate for doctors to remain in control of decisions about ending pregnancies in light of AWs? AWT has the potential to alleviate women of the burdens placed solely on them in reproduction, but this is only possible if women are empowered to decide which risks are tolerable. It is possible that healthcare policy might still present high hurdles in terms of access to this technology in future; however, it is not appropriate for the criminal law to impose heteronormative conceptions of the female body and its role in gestation.

## Footnotes

1 E Partridge and others, ‘An Extra-uterine System to Physiologically Support the Extreme Premature Lamb’ (2017) 8 Nature Communications 1, 11; H Usuda and others, ‘Successful Use of an Artificial Placenta to Support Extremely Preterm Ovine Fetuses at the Border of Viability’ (2019) American Journal of Obstetrics and Gynecology*.* doi: https://doi.org/10.1016/j.ajog.2019.03.001.

2 The AWs from recent experiments are designed with the intention of facilitating the continued gestation of developing human entities delivered from a pregnant woman’s uterus prematurely. Usuda and others (n 1) are explicit that the object of their AW device is to provide an environment in which the subject can behave ‘more like a foetus than a baby’.

3 Partial ectogenesis is the development of a human being in an AW for part of the typical gestational period following transfer from the maternal womb. C Kaczor, *The Edge of Life: Human Dignity and Contemporary Bioethics, Philosophy and Medicine* (1st edn, Springer 2005), 113.

4 EC Romanis, ‘Artificial Womb Technology and the Frontiers of Human Reproduction: Conceptual Differences and Potential Implications’ (2018) 44 Journal of Medical Ethics 751.

5 This burden is placed on any person with the reproductive biology that allows them to become pregnant (female) irrespective of gender identity. In this article, I refer to women and to pregnant women because the vast majority of people who experience pregnancy identify as women, and this massively impacts on the social experience of pregnant people and the social and medical response to them.

6 Hendricks posits that AWs could reduce the risks to health incumbent on women in pregnancy. Smajdor argues that AWs are a moral imperative because they will alleviate women from the burdens and suffering innate in pregnancy. See J Hendricks, ‘Not of Woman Born: A Scientific Fantasy’ (2011) 62 Case Western Reserve Law Review 399, 408–09; A Smajdor, ‘The Moral Imperative for Ectogenesis’ (2007) 16 The Cambridge Quarterly of Healthcare Ethics 336, 337. Kendal posits that AWs are a ‘much needed option for those women who wish to have children without submitting to the physical burdens of gestation and childbirth…’ and to potentially evade some of the ‘social burdens’ associated with gestation. See E Kendal, *Equal Opportunity and the Case for State Sponsored Ectogenesis* (Palgrave 2015) 8.

7 ibid.

8 These important issues related to clinical translation are explored in EC Romanis, ‘Artificial Womb Technology and Clinical Translation: Innovative Treatment or Medical Research?’ (2019) Bioethics; doi: 10.1111/bioe.12701.

9 Hendricks (n 6); Smajdor (n 6); Kendal (n 6).

10 A Grubb, ‘Abortion Law in England ‘(1990) 18 Law, Medicine and Healthcare 146, 156.

11 Issues related to speculative discourse are addressed in ongoing co-authored research with Claire Horn at Birkbeck, University of London.

12 Eg S Sheldon, *Beyond Control: Medical Power and Abortion Law* (Pluto Press 1997); E Jackson, *Regulating Reproduction: Law, Technology and Autonomy* (1st edn, Hart Publishing 2001) 80; S Sheldon, ‘Abortion Act 1967: A Critical Perspective’ in E Lee (ed), *Abortion Law and Politics Today* (1st edn, Macmillian Press Ltd 1998).

13 J Dalzell, ‘The Impact of Artificial Womb Technology on Abortion Jurisprudence’ (2019) 25 William & Mary Journal of Race, Gender and Social Justice 327; J Schultz, ‘Development of Ectogenesis: How will Artificial Wombs Affect the Legal Status of a Fetus or Embryo’ (2010) 84 Chicago-Kent Law Review 877; A Alghrani, ‘Viability and Abortion: Lessons from Ectogenesis,’ (2009) 4 Expert Review of Obstetrics and Gynecology 625.

14 C Overall, ‘Rethinking Abortion, Ectogenesis and Foetal Death’ (2015) 46 Journal of Social Philosophy 126; J Räsänen, ‘Ectogenesis, Abortion and a Right to the Death of the Fetus’ (2017) 31 Bioethics 697; P Hendricks, ‘There is No Right to the Death of the Fetus’ (2018) 32 Bioethics 395.

15 I use the term ‘conventional abortion’ to refer to the medical process of terminating a pregnancy that results in foetal death. Conventional abortions include drug-induced abortion and surgical abortion.

16 A Alghrani, ‘Regulating the Reproductive Revolution: Ectogenesis – A Regulatory Minefield?’ in M Freeman (ed), *Law and Bioethics: Volume 11* (1st edn, OUP 2008) 316.

17 Department of Health, ‘Abortion Statistics, England and Wales 2017’ (2018) 4 <https://www.gov.uk/government/statistics/abortion-statistics-for-england-and-wales-2017> (accessed 10 August 2018).

18 The account given as to why AWT is not an alternative to conventional abortion has been greatly influenced by research collaboration with Claire Horn at Birkbeck, University of London. Also see S Langford, ‘An End to Abortion? A Feminist Critique of the “Ectogenetic Solution” to Abortion’ (2008) 31 Women’s Studies International Forum 263.

19 L Cannold, ‘Women, Ectogenesis and Ethical Theory’ (1995) 12 Journal of Applied Philosophy 55.

20 There might be future calls to attempt to use the current prototype AW devices to develop *complete* ectogenesis whereby embryos could be gestated entirely *ex utero* (without the need for a pregnancy). The prospects of AWT being realistically used for this process are far less likely. Complete ectogenesis is still decades away, because of the legal and political realities limiting embryo research, and because of the uncertain realities of embryo science. The technology we have, at least for now, is only a viable facilitator of partial ectogenesis (rather than eliminating the need for all pregnancy).

21 See n 5.

22 The Offences Against the Person Act 1861 also criminalised termination of pregnancy in Northern Ireland. However, a majority of the House of Commons voted to decriminalise termination in Northern Ireland in early July 2019. Abortion has been decriminalised since the repealing of the law in Northern Ireland (Executive Formation) Act 2019, s 9 came into effect on 22 October 2019. The Government has now launched a consultation about what regulatory framework should be put in place concerning abortion provision. See HM Government, ‘A New Legal Framework for Abortion Services in Northern Ireland’ <https://assets.publishing.service.gov.uk/government/uploads/system/uploads/attachment_data/file/844394/Government_consultation_-__A_new_legal_framework_for_abortion_services_in_Northern_Ireland__November_2019_.pdf> (accessed 7 November 2019).

23 Offences Against the Person Act 1861, ss 58 and 59.

24 ibid.

25 ibid, s 58.

26 ibid.

27
*R (On the Application of Smeaton) v Secretary of State for Health* [2002] EWHC 610 (admin) (Munby J) para 17.

28 A Alghrani and M Brazier, ‘What Is It? Whose Is It? Re-positioning the Foetus in the Context of Research?’ (2011) 70 Cambridge Law Journal 51, 75.

29 The biobag has also been referred to as ‘EXTEND’ therapy in more recent publications by this research team. See A Rossidis and others, ‘Premature Lambs Exhibit Normal Mitochondrial Respiration after Long-Term Extrauterine Support’ (2019) Fetal Diagnosis and Therapy. doi: 10.1159/000496232.

30 Partridge and others (n 1).

31 Note that this research group first published results in 2017, but published results of a refined device in 2019: H Usuda and others, ‘Successful Maintenance of Key Physiological Parameters in Preterm Lambs Treated with Ex vivo Uterine Environment Therapy for a Period of 1 week’ (2017) 217 American Journal of Obstetrics and Gynaecology 1; Usuda and others (n 1).

32 A gestateling is ‘a human being in the process of ex utero gestation exercising, whether or not it is capable of doing so, no independent capacity for life’: Romanis (n 4) 753. The term gestateling will be used throughout this article to refer to a human being in the process of gestation *ex utero*. A unique term is used because the subject of an AW is conceptually distinct from both a foetus and a neonate.

33 Romanis (n 4) 753.

34 K Azad and J Matthews, ‘Preventing Newborn Deaths Due to Prematurity' (2016) 36 Best Practice & Research Clinical Obstetrics and Gynecology 131, 132.

35 In particular infants born at or before 24 weeks often have lungs too immature to withstand pulmonary gas exchange, and thus cannot acquire sufficient oxygen.

36 T Lissauer and G Clayden (eds), *Illustrated Textbook of Pediatrics* (4th edn, Mosby Elsevier 2012) 164.

37 ibid 159.

38 ibid 167.

39 Partridge and others (n 1) 6.

40 ibid 2, 6.

41 The EVE platform study has a lower survival rate (of 87.5%) and incidences of white matter brain injury and early signs of liver dysfunction amongst trial subjects (lamb foetuses): Usuda and others (n 1).

42 Usuda and others (n 1).

43 ibid 10.

44 J Couzin-Frankel, ‘Fluid-filled ‘biobag’ Allows Premature Lambs to Develop Outside the Womb’ (*Science*, 25 April 2017) <http://www.sciencemag.org/news/2017/04/fluid-filled-biobag-allows-premature-lambs-develop-outside-womb> (accessed 11 October 2017). Dr Flake, leading the biobag study, suggests that even only a decade from now AWT could have completely replaced NIC: Children’s Hospital of Philadelphia, ‘Unique Womb-like Device Could Reduce Mortality and Disability for Extremely Premature Babies’, 25 April 2017 <https://medicalxpress.com/news/2017-04-unique-womb-like-device-mortality-disability.html?utm_source=TrendMD&utm_medium=cpc&utm_campaign=MedicalXpress_TrendMD_1> (accessed 1 April 2019).

45 Partridge and others (n 1) 11.

46 Alghrani (n 16) 307.

47 This point is identified because it is the age of the youngest premature neonate that has ever survived. It is notable, however, that lung development will occur at a slightly different rate for different foetuses and so this is not a fixed point.

48 This approach was actually recommended by the Nuffield Council on Bioethics in their 2006 Report. See Nuffield Council on Bioethics, *Critical Care Decisions in Fetal and Neonatal Medicine: Ethical Issues* (2006) <http://nuffieldbioethics.org/wp-content/uploads/2014/07/CCD-web-version-22-June-07-updated.pdf> (accessed 27 April 2018).

49 The exact point of foetal viability is disputed, but there is broad consensus that lung maturity is the deciding factor. Whilst there are some examples of foetuses having survived before 22 weeks, the vast majority of foetuses born before this point do not have solid lungs. This viability is usually estimated to be between 22 and 24 weeks.

50 Romanis (n 4) 752.

51 Alghrani and Brazier point out that ‘once the foetus is ex utero, alive or dead, and once a woman’s bodily integrity is no longer at stake, the male progenitor should have an equal say with regard to foetal treatment and paternal consent should also be sought’. See Alghrani and Brazier (n 28) 62.

52 Romanis (n 4) 753.

53 Alghrani and Brazier identify this point as a meaningful point to engage with a shifting viability threshold and the law, thus I also adopt this point in this article. See Alghrani and Brazier (n 28) 62.

54 Partridge and others (n 1).

55 I Symonds, P Baker and L Kean, *Problem Orientated Obstetrics and Gynaecology* (1st edn, Arnold Publishers 2002) 302.

56 Where the placenta becomes detached from the uterine wall and compromises the foetal condition: ibid 103. During or following placental abruption, terminating the pregnancy is the only chance to save the life of the pregnant woman, and depending on the duration of gestation, her foetus.

57 If a pregnant woman suffers a severe traumatic injury, their prognosis, and that of the foetus when their vital signs are weak or absent, are often improved by terminating the pregnancy preterm. See C Beckman and others, *Obstetrics and Gynaecology* (7th edn, Wolters Kluwer 2014) 203.

58 Preeclampsia is a serious condition characterised by high blood pressure, which if unmanaged leads to organ damage, neurological symptoms and fluid in the lungs. Substances being released from the placenta into the maternal blood vessels cause the condition to develop. See C Nierenberg, ‘Preeclampsia: Signs, Symptoms & Treatment’ (*Live Science*, 01 June 2017) <https://www.livescience.com/51401-what-is-preeclampsia.html> (accessed 10 August 2018). Preeclampsia occurs in between 2 and 4% of pregnancies (Symonds, Baker and Kean (n 55) 52), and usually necessitates premature termination of pregnancy. Once preeclampsia is diagnosed, delaying delivery only prolongs the risk to the pregnant woman’s long-term health and life (Symonds, Baker and Kean (n 55) 55). By remaining pregnant, the woman risks seizures and long-term impaired liver and kidney function.

59 Chronic high blood pressure occurs in around 1–2% of all pregnancies and increases the likelihood of developing preeclampsia and/or placental abruption necessitating premature termination. Premature delivery is often recommended to manage these risks. See Symonds, Baker and Kean (n 55) 50.

60 Approximately 0.5% of pregnant women are diabetic and gestational diabetes is developed in a further 3% of pregnancies. See Symonds, Baker and Kean (n 55) 37. Diabetes increases the risk of preeclampsia, foetal compromise, or birth trauma and, consequently, premature terminations of pregnancy are often recommended to manage risks: Symonds, Baker and Kean (n 55) 40.

61 Unmanaged uterine infection, causing inflammation of the pelvis, occurs in 2% of pregnancies (Beckman and others (n 57) 201) and increases the likelihood that the pregnancy will need to be ended before the end of the gestational period to better treat the pregnant woman.

62 For example, when there is a reduction in amniotic fluid or the foetal heart rate is weak or abnormal. See Symonds, Baker and Kean (n 55) 84–85.

63 When there is intrauterine growth restriction folds, early termination of pregnancy is often advised. See Symonds, Baker and Kean (n 55) 90.

64 EM Symonds and I Symonds, *Essential Obstetrics and Gynaecology* (4th edn, Elsevier Science 2002) 107.

65 Common risks associated with premature delivery for the foetus include, but are not limited to, underdeveloped lungs and respiratory issues, circulatory problems, low blood pressure, and an inability to swallow or suck. See Lissauer and Clayden (n 36) 164, 167.

66 For example infection, see ibid 164.

67 For further discussion of the conceptual differences between AWs and conventional neonatal intensive care, see Romanis (n 4).

68 Symonds, Baker and Kean (n 55) 55.

69 Lissauer and Clayden (n 36) 159.

70 In some instances, when complications and the need for premature termination of pregnancy become evident earlier in the pregnancy, steroids can be administered to accelerate lung maturity to reduce some of the most significant risks associated with preterm birth. See Symonds, Baker and Kean (n 55) 90.

71 National Institute for Health and Care Excellence, ‘Information for the Public; Caesarean Section’ (2011) 3 <http://publications.nice.org.uk/ifp132> accessed 11 August 2018.

72 There are several different pharmaceuticals that can be used to induce delivery, for example, oxytocins or prostaglandins: see Symonds, Baker and Kean (n 55) 71, 125.

73 For example, caesarean sections heighten the risk of infection, excessive blood loss and blood clots and the use of anaesthesia can be dangerous for some women. See National Institute for Health and Care Excellence (n 71) 12–15. Drug-induced delivery can cause foetal hypoxia if the uterus fails to sufficiently relax. See Symonds, Baker and Kean (n 55) 124.

74 In instances where a pregnant woman faces a threat to her own health, but wants to give her foetus the best chance of life, this can be a very difficult decision to make: AWT might effectively make it so that pregnant women do not have to actively make a choice between their health and their foetuses.

75 In 2016, Heidi Loughlin was diagnosed with an aggressive cancer whilst pregnant and was forced to choose between life-saving treatment and remaining pregnant. This case caught the attention of the national media. Heidi opted for premature delivery at 28 weeks to receive treatment in the hope that her preterm daughter would be developed enough to successfully be supported by NIC. Waiting this long for treatment was extremely risky for Heidi because the cancer continued to grow and spread during the time she remained pregnant. She fortunately survived, however her neonate, Ally Loughlin, died soon after delivery: P Walker, ‘Baby Delivered Early to Allow Mother’s Cancer Treatment Dies’ *The Guardian* (London, 20 December 2015) <https://www.theguardian.com/uk-news/2015/dec/20/baby-delivered-early-to-allow-mothers-cancer-treatment-dies> accessed15 April 2019.

76 By this phrase I mean to suggest that foetuses could be considered viable if they were placed in a machine able to continue gestation, but they are not ‘naturally viable’ because they have not reached sufficient developmental maturity to survive without gestational support aiding continued organ maturation.

77 The ‘maternal-foetal conflict’ model is a way of conceptualising pregnancy that presents the interests of the pregnant woman and foetus as in contention with each other. Bowden presents a compelling argument, even outside of the possibility of technology like AWT, as to why there is an inherent problem in adopting this conceptualisation, because the welfare of a pregnant woman and her foetus are frequently interconnected. See C Bowden, ‘Are We Justified in Introducing Carbon Monoxide Testing to Encourage Smoking Cessation in Pregnant Women?’ (2019) 27 Health Care Analysis 128, 139.

78 O Keag, J Norman, and S Stock, ‘Long Term Risks and Benefits Associated with Caesarean Delivery for Mother, Baby and Subsequent Pregnancies: Systematic Review and Meta-analysis’ (2018) 15(1) PLOS Medicine e1002494.

79 Kendal (n 6) 12–13.

80 In using these examples, I am not trying to suggest that women in these circumstances should seek AWs in place of conventional termination or remaining pregnant. I only wish to suggest that, in those instances where a woman wants to become a mother or wants to give up her foetus for adoption (rather than pursue abortion for their own reasons), partial ectogenesis could be a favourable option for someone with those preferences in some circumstances.

81 The law is clear that, outside of emergencies, patients do not have the right to demand any particular treatment over another following *R (Burke) v General Medical Council* [2005] EWCA Civ 1003. This is evident in the context of prenatal care: for example, while the law is supportive of choice in childbirth a woman cannot demand one form of childbirth over another, eg an elective caesarean, if their doctor is unwilling to offer that choice. See EC Romanis, ‘Why the Elective Caesarean Lottery is Ethically Impermissible’ (2019) Health Care Analysis. doi: 10.1007/s10728-019-00370-0.

82 ibid; also the law is supportive of doctors’ right to conscientiously object to certain treatments in a number of instances. In this context, it is notable that doctors are entitled to object, on the grounds of conscience, to provide pregnancy terminations when the termination is not necessary to save the life of the pregnant woman. See Abortion Act 1967, s 4.

83 There are interesting socio-political implications associated with the increasing availability of AWs—for example, the negative implications of women feeling the need to opt out of complete pregnancy in order to return to work earlier, or the use of AWs to police pregnancy where women are non-compliant eg drug users. Such issues are beyond the scope of this article.

84 N Hammond-Browning, ‘A New Dawn: Ectogenesis, Future Children and Reproductive Choice’ (2018) 14 Contemporary Issues in Law 349, 365.

85 ibid 362

86 Kendal (n 6) 8, 12–13. I agree that ectogenesis could present an important reproductive choice for women; however, it is inappropriate to label ectogenesis as a solution to social problems since this suggests that there *is* something inferior about the female body and its role in gestation. The argument has the implication that we should remove gestation from the body to solve discrimination against women, rather than attempt to deconstruct the problematic social reactions to gestation and the female body. Claire Horn and I develop this argument in our ongoing research.

87 See the text accompanying nn 25 and 26 above.

88 Offences Against the Person Act 1861, ss 58 and 59.

89
*Smeaton* (n 27) para 338.

90 B Dickens, *Abortion and the Law* (1st edn, MacGibbon & Kee 1966) 25.

91 V Harpwood, *Legal Issues in Obstetrics* (1st edn, Dartmouth 1996) 216.

92 Dickens (n 90) 30.

93 ibid.

94
*Smeaton* (n 27) para 17 (emphasis added).

95 Harpwood (n 91) 216.

96
*Smeaton* (n 27).

97 ibid, para 17.

98 E Martin, *Concise Medical Dictionary* (9th edn, OUP 2015) 480–81; M Page (ed), *British Medical Association Illustrated Medical Dictionary* (3rd edn, Dorling Kindersley 2013) 370.

99 Under this interpretation (miscarriage as termination resulting in foetal death), there would be no offence even where the foetus dies (despite best efforts), so long as there was no intention that it would not survive the ending of the pregnancy, because the mens rea of the offence would be ‘an intention to procure a termination of pregnancy resulting in the death of the foetus’. In some instances, there might be considered to be an oblique or indirect intention if it could be demonstrated that the doctor ending the pregnancy was ‘virtually certain’ that the foetus would die as a result of their actions, to such an extent that they can be said to have intended that outcome.

100 Infant Life (Preservation) Act 1929, s 1.

101 ibid.

102 Grubb (n 10) 149.

103 ibid 149.

104 M Brazier and J Harris, ‘"Fetal Infants:” At the Edge of Life’ in P Ferguson and G Laurie (eds), *Inspiring a Medico-Legal Revolution; Essays in Honour of Sheila McLean* (1st edn, Ashgate Publishing 2015) 55.

105 Alghrani and Brazier (n 28) 75.

106 Offences Against the Person Act 1861, s 59.

107 ibid, ss 58 and 59.

108 An unborn foetus has never been considered a person for the purposes of the law of homicide see: *R v Senior* (1832) 1 Mood. C.C. 346.

109
*Paton v British Pregnancy Advisory Service* [1979] QB 276.

110 See the text accompanying n 26.

111 As in *R v Bourne* [1939] 1 KB 687.

112 W Chan and AP Simester, ‘Four Functions of Mens Rea’ (2011) 70 Cambridge Law Journal 381.

113 Some of the most common pharmaceuticals inducing delivery, prostaglandins, were not discovered until 1976.

114 United States National Library of Medicine, ‘Cesarean Section – A Brief History’ (2018) <https://www.nlm.nih.gov/exhibition/cesarean/part2.html> accessed 01 August 2018.

115 The development of a neonatal intensive care unit is attributed to Dr. Martin Couney. He first exhibited a design at the World Exposition in Berlin in 1896. See W Silverman, ‘Incubator-Baby Side Shows’ (1979) 64 Pediatrics 127, 129.

116 In the *Smeaton* judgment, evidence from medical textbooks of the period when the OAPA was drafted was considered as an aid to interpreting the meaning of miscarriage. In evidence submitted there was the implication that premature labour was something spontaneous. See *Smeaton* (n 27) para 157; J Burns, *The Principles of Midwifery including the Diseases of Women and Children* (10th edn, Benjamin Warner, Edward Parker, Mathew Carey & Son, Benjamin & Thomas Kite, Solomon W. Conrad, and Anthony Finley 1843) 304–05.

117
*Smeaton* (n 27) para 337.

118 ibid.

119 ibid, para 329.

120 ibid, para 334.

121 ibid, para 350.

122 [1998] AC 147.

123 ibid (Lord Steyn) 156.

124 G Slapper and D Kelly, *The English Legal System* (13th edn, Routledge 2012) 103.

125 See deference to medical opinion in the judgments of: *Paton* (n 109), and *Bourne* (n 111).

126 Martin, *Concise Colour Medical Dictionary* (6th edn, OUP 2015) 480.

127 R Pease (ed), *Merriam-Webster’s Medical Dictionary* (1st edn, Merriam-Webster 1995) 2.

128 H Marcovitch (ed), *Black’s Medical Dictionary* (43rd edn, Bloomsbury 2017).

129
*Smeaton* (n 27) para 157; Burns (n 116).

130 See *Heydon’s case* (1584) EWHC Exch J36.

131
*Smeaton* (n 27) para 354.

132 Symonds and Symonds (n 64) 303.

133
*Smeaton* (n 27) para 354.

134 ibid, para 354.

135 There are political complications to the decriminalisation debate; however, the developments referred to in n 22 do demonstrate that it is politically possible.

136 There are good reasons to question the legitimacy and ethics of criminalisation: E Jackson, ‘Abortion, Autonomy and Prenatal Diagnosis’ (2000) 9 Social & Legal Studies 467. For the current campaign for decriminalisation of abortion in the UK, see ‘We Trust Women’ <https://www.wetrustwomen.org.uk/5-reasons-to-decriminalise-abortion/> accessed 24 May 2019.

137 Here I make no comment on the ethics of conventional abortion as I have already advocated that AWs should not be thought of as an alternative to conventional abortion and, thus, AWs should not be used as grounds to limit abortion access. Here, I am only attempting to delineate conventional abortion from termination to continue gestation *ex utero* to suggest that even if regulation of conventional abortion is appropriate on the grounds of some ‘state interest’ in the foetus, this same reasoning should not be used to regulate terminations not intended to result in foetal death.

138 A Grubb, ‘The New Law of Abortion: Clarification or Ambiguity?’ (1991) Criminal Law Review 659, 665.

139 The stigma automatically attached to ‘criminal termination of pregnancy’ is another good reason to support decriminalisation of abortion. See R Cook, ‘Stigmatized Meanings of Criminal Abortion Law,’ in R Cook, J Erdman and B Dickens (eds), *Abortion Law in Transnational Perspective: Cases and Controversies* (1st edn, University of Philadelphia Press 2014).

140 Abortion Act 1967, s 1 (1), as amended by Human Fertilisation and Embryology Act 1990, s 37.

141 ibid, s 1 (1) (b) and (c), as amended by Human Fertilisation and Embryology Act 1990, s 37.

142 The defence of necessity is likely to have applied. See *Bourne* (n 111).

143
*Re T (Adult: Refusal of Treatment)* [1993] Fam 95 (Lord Donaldson) 102.

144 The language of ‘state interest in fetal life’ is explicit in US law following *Roe v Wade* (1973) 410 US 113 (United States Supreme Court). In the UK a similar approach is adopted (though less explicitly justified in terms of ‘state interest’), because a foetus is afforded more protection in law from 24 weeks gestation when it is deemed ‘capable of being born alive’: Infant Life (Preservation) Act 1929, s 1 (1).

145 Abortion Act 1967, s 1 (1) (a), as amended by Human Fertilisation and Embryology Act 1990, s 37.

146
*Bourne* (n 111).

147 ibid (Macnaghten J) 691.

148 Abortion Act 1967, s 1 (1) (c), as amended by Human Fertilisation and Embryology Act 1990, s 37.

149 [1909] 2 Sask LR 46 (Supreme Court of Saskatchewan).

150 The Criminal Code 1892, s 303 (Canada). This version of the Criminal Code has since been repealed and replaced.

151
*Re McCready* (n 149) (Lamont J) para 9.

152 ibid, para 9.

153 ibid, para 10.

154
*Bourne* (n 111).

155 Dickens (n 90) 39.

156 The ‘social’ ground for abortion in Abortion Act 1967, s 1 (1) (a), as amended by Human Fertilisation and Embryology Act 1990, s 37, provides a defence for all terminations carried out before 24 weeks where the risk of continuing the pregnancy is greater than the risk of terminating the pregnancy. This is easily established today in the vast majority of pregnancies, given the availability of abortifacient drugs carrying far fewer risks than surgical abortions and than remaining pregnant. See M Brazier and E Cave, *Medicine, Patient’s and the Law* (6th edn, Manchester UP 2016) 404.

157 J Mason and G Laurie, *Mason and McCall Smith’s Law and Medical Ethics* (9th edn, OUP 2013) 336.

158 Jackson (n 12) 80.

159 Brazier and Cave (n 156) 404.

160 J Murphy, ‘Is Pregnancy Necessary? Feminist Concerns about Ectogenesis’ in S Gelfand and J Shook (eds), *Ectogenesis: Artificial Womb Technology and the Future of Human Reproduction* (1st edn, Rodopi 2006) 34; Schultz (n 13) 880.

161 J Mason, *The Troubled Pregnancy* (1st edn, CUP 2007) 25.

162 Abortion Act 1967, s 4.

163
*Burke* (n 81).

164 Abortion Act 1967, s 1 (1) (c), as amended by Human Fertilisation and Embryology Act 1990, s 37.

165 ibid, s 1 (1) (b).

166 ibid, s 1 requires that two medical professionals form this opinion; however, if a medical professional forms the opinion in good faith that termination of pregnancy is immediately necessary to save the pregnant woman’s life or to prevent serious injury to their health, the medical practitioner may act alone. See Brazier and Cave (n 156) 404.

167 Grubb (n 138) 661.

168 This will be explored in detail later in this article. For now, it is sufficient to note that if doctors are concerned that pregnancy poses a risk to life, they can act when that risk is greater than the risk of termination. Yet when doctors are concerned only about the impact of pregnancy to health (but not life), that impact on health must be ‘grave’ or ‘permanent’. Thus, there are more qualifications on the risk to health than potential risks to life.

169 This was established in *Bourne* (n 111), a case that considered the legality of termination of pregnancy before the AA 1967; D Morgan and RG Lee, *Blackstone’s Guide to the Human Fertilisation and Embryology Act 1990* (1st edn, Blackstone Press 1990) 51.

170 Abortion Act 1967, s 1 (1) (c), as amended by Human Fertilisation and Embryology Act 1990, s 37.

171 Harpwood (n 91), 231.

172 For example, when a woman is suffering from a severe traumatic injury, preeclampsia, or gestational diabetes.

173 If a woman has preeclampsia, for example, regardless of when the ending of the pregnancy takes place, it is safest for the product of pregnancy to be removed by caesarean section. Therefore, the risks of a caesarean are inherent to that pregnancy whenever it is ended. These risks thus become almost irrelevant for the purposes of the defence in Abortion Act 1967, s 1 (1) (a), as amended by Human Fertilisation and Embryology Act 1990, s 37.

174 There might be questions about whether this defence technically applies in some instances in which ‘premature delivery’ is routinely performed—for example, when termination is performed to extract a foetus with stunted growth.

175 This is obviously dependent on the pregnant woman’s particular circumstances. A pregnant woman with very severe nausea who risks developing kidney problems, for example, could argue there could be some risk to their life.

176 Jackson (n 12) 78.

177 Harpwood (n 91) 228.

178 As amended by Human Fertilisation and Embryology Act 1990, s 37.

179 Morgan and Lee (n 169) 50.

180 ibid 52.

181 Harpwood (n 91) 231.

182 Morgan and Lee (n 169) 50.

183 E Jackson, ‘Abortion’ in I Kennedy and others (eds), *Principles of Medical Law* (4th edn, OUP 2017) 867.

184 HL Vol 522 Col 1039.

185 Morgan and Lee (n 169) 51.

186 HL Vol 522 Col 1039.

187 ibid.

188 In these instances a caesarean delivery cannot be performed as, once the foetus is not fully in the uterus, it is too late to perform the procedure, and the foetus is stuck in the birth canal.

189 An explicit attempted amendment in the House of Lords to stipulate that later terminations when the foetus may be ‘capable of being born alive’ should mean that the medical practitioner is required to take ‘all reasonable steps to procure that the child is born alive’ was defeated in a vote. See HL Vol 522 Cols 1043–1087.

190 Pregnancy forces a woman to share her bodily resources, literally stripping her of nutrients and draining her of the energy needed to conduct daily activities. The occupation of her body causes her to experience severe hormonal and chemical changes that affect how she feels, behaves, and perceives the world. Changes to physical appearance necessarily affect how a pregnant woman is treated. Lots of this cannot be undone. After 9 months her body is (often) torn apart by the process of childbirth and there will be permanent scars, and even damage. Even following the pregnancy, she faces serious risks associated with it: for example, there is the risk of experiencing post-partum depression and suicide. This is, of course, not to say that all experiences of pregnancy are negative, but it is important that we acknowledge some of the negative experiences often innate to an individual pregnancy.

191
*Bourne* (n 111) (Macnaghten J) 694.

192 Jackson (n 12) 78.

193
*Bourne* (n 111).

194 ibid (MacNaughten J) 694.

195 ibid.

196 Macnaghten J commented that the doctor’s decision, ‘you will observe … has nothing to do with the ordinary case of procuring abortion to which I have already referred’. ibid (Macnaghten J) 691.

197 J Montgomery, ‘Medicine, Accountability and Professionalism’ (1989) 16 Journal of Law and Society 319, 325.

198
*Paton* (n 109).

199 ibid (Baker P) 282.

200 [1973] 1 WLR 1510.

201 ibid (Scarman LJ) 1516.

202 J Keown, *Abortion, Doctors and the Law: Some Aspects of the Legal Regulation of Abortion in England and Wales from 1803 to 1982* (1st edn, CUP 1988) 165.

203
*R v Misra and Srivastava* [2005] 1 Cr App R 328.

204 EC Romanis, ‘Challenging the ‘Born Alive’ Threshold: Foetal Surgery, Artificial Wombs and the English Approach to Legal Personhood’ (2019) *Medical Law Review*, online first doi: https://doi.org/10.1093/medlaw/fwz014.

205 S Sheldon, ‘The Medical Framework and Early Abortion in the UK: How Can a State Control Swallowing?’ in R Cook, J Erdman and B Dickens (eds), *Abortion Law in Transnational Perspective* (University of Pennsylvania Press 2014) 207

206 ibid 207.

207 Unfortunately, there has only been limited scope in this article to explain why this argument should also apply to conventional abortion.

208
*Bourne* (n 111).

